# Association of sarcopenia with survival and treatment response in brain metastasis of non-small cell lung cancer

**DOI:** 10.1038/s41598-026-37138-1

**Published:** 2026-02-05

**Authors:** Leon Schmidt, Harald Krenzlin, Anika Schmitz, Dragan Jankovic, Alice Dauth, Beat Alessandri, Clemens Sommer, Marc A. Brockmann, Florian Ringel, Naureen Keric

**Affiliations:** 1 Department of Neurosurgery, University Medical Center Mainz, Mainz, Germany; 2 Department of Neurosurgery, University Hospital Schleswig-Holstein (UKSH), Ratzeburger Allee 160, 23538 Lübeck, Germany; 3 Institute of Neurpathology, University Medical Center Mainz, Mainz, Germany; 4 Department of Neuroradiology, University Medical Center Mainz, Mainz, Germany

**Keywords:** Brain metastasis, Lung cancer, Immunotherapy, Frailty, Sarcopenia, Surgical oncology, Neurology

## Abstract

Brain metastases are common in non-small cell lung cancer (NSCLC) and affect prognosis and survival. While frailty and sarcopenia are associated with the overall survival in NSCLC the impact on outcome and survival after surgery for brain metastasis is unknown. We therefore analyzed 179 patients (81 women) with NSCLC undergoing resection for brain metastasis between 2011 and 2020 retrospectively. Frailty was measured using the Clinical Frailty Scale (CFS). Temporal Muscle Volume (TMV) was assessed in preoperative T1w MRI. The median age was 63 years. Clinical frailty was present in about 20.6%. Mean follow-up was 11 months. Frailty correlated significantly with age (*r* = 0.36, *p* < 0.001) and smaller TMV (*r*=-0.24, *p* = 0.002). However, only measurement of TMV predicted impaired survival (median OS 34.5 vs. 10.3 months, *p* < 0.001). Physical performance after surgery was negatively affected by frailty (*r*=-0.72, *p* < 0.001) and positively by TMV (*r* = 0.2, *p* = 0.038). Major postoperative complications were more strongly associated with sarcopenia rather than frailty. Treatment response towards immunotherapy improved in the absence of sarcopenia (B = 2.48, *p* = 0.031). TMV is a predictor for survival after resection of brain metastasis and an indicator of treatment response to immunotherapy in patients with NSCLC. Accounting for sarcopenia in surgical decision making could improve patient selection for different treatment modalities.

## Introduction

 Lung cancer is the second most common malignancy with an estimated yearly incidence of 2,206,771 cases worldwide in 2020 and accounts for 18% of cancer related deaths^1^.

In NSCLC brain metastases are frequent with 20% of all patients suffering from CNS dissemination. Brain metastases cause substantial disability, reduced quality of life and impaired prognosis^2^. The cumulative incidence rises throughout the course of the disease^3,4^. Lung cancer is largely diagnosed in older age^5^. Frailty, the clinical state of increased vulnerability and susceptibility to disease resulting from aging and age associated decline is known to influence therapy response and survival^6–8^. Although the prevalence of frailty increases with age, chronological age alone fails to sufficiently address these multifactorial changes^9^. Thus frailty has become a valuable predictor of outcome and mortality independent from biological age^10–13^. Frailty is prevalent in 45% of patients suffering from NSCLC and increases the mortality risk threefold^13^. It is associated with lower overall survival (OS) in early-stage NSCLC with patients more likely to die of causes unrelated to the primary malignancy^14^. Independent from NSCLC, it has been established that frailty poses an increased risk of postoperative complications, chemotherapy intolerance, disease progression, and death^23^. In the context of oncological surgery frailty is associated with an increased risk for major complications in most solid malignancies^8,15–19^.

Rather than age, frailty includes different characteristics, including sarcopenia. According to international recommendations, sarcopenia is defined by low muscle strength and quantity. Muscle mass can be assessed using CT or MRI^20^. In the context of neurological illnesses, temporal muscle assessment correlates closely with sarcopenia^21,22^. Advantages for the clinical application in neuro-oncologic surgery arise from the availability in this patient collective, while whole-body imaging might not be readily available in the initial setting when key therapy decisions are made.

Sarcopenia is an established predictor of impaired survival in lung cancer and other malignancies^23–28^. It increases the risk of all types of complications in patients undergoing oncological surgery^27^. However, data in the context of neuro-oncologic surgery is sparse. In the therapy of brain metastasis immunotherapy is of increasing relevance, but most data is derived from younger and fitter patients, not the older and frail^29^. The current data suggests that safety and tolerability of checkpoint inhibitors is similar, while the impact of adverse events in those with frailty increased greater than in younger and fit patients^30,31^. In general, patients affected by frailty show impeded response to immunotherapy while being at an increased risk of suffering toxicity and complications jeopardizing treatment success and adjusted therapy planning^10,12,32,33^. The relevance of frailty and sarcopenia for resection and survival in patients with NSCLC and brain metastasis is still unknown. Validated cut-off values defining sarcopenia as a marker of frailty in patients with NSCLC and brain metastasis are missing. It is the aim of this study to establish the impact of frailty and sarcopenia on surgical treatment and immunotherapy in patients with NSCLC and brain metastasis.

## Methods

For the presented study we included patients who underwent resection for cerebral or cerebellar metastasis of non-small-cell lung cancer (NSCLC) at a single institution from 2011 to 2020. The present study follows the STROBE guidelines for cohort studies.

### Patients

Patients were required to be over 18 years of age and to have undergone resection of cerebral or cerebellar NSCLC metastasis. All patients received tumor resection of at least one lesion. Progression free survival (PFS) and overall survival (OS) measured in months were defined from surgery until radiological progression or death respectively. Postoperative complications were defined as the emergence of pneumonia, pulmonary embolism (PE), deep-vein thrombosis (DVT), surgical site infection or rebleeding during the in-patient treatment period. Presence of any of these complications was termed combined as composite complications. During institutional multidisciplinary tumor board meetings, treatment decisions concerning the surgical procedure and adjuvant treatment were made prior to and after surgery.

To compare TMV values with a previously healthy cohort we included 31 patients who were treated at our department suffering from aneurysmal subarachnoid hemorrhage between January 2016 and December 2020. Patients were included if an MRI was performed within 14 days after hemorrhage to reduce the chance of bed-rest induced atrophy after critical illness.

### Clinical scores

To evaluate patients according to their frailty and functional status we utilized the Karnofsky Performance Score (KPS), Clinical Frailty Scale (CFS), and Charlson Comobidity Index (CCI). The KPS quantifies the functional status of cancer patients on a scale from 0 (dead) to 100 (normal no complaints; no evidence of disease)^34^. The Clinical Frailty Scale is a common scoring method to evaluate frailty ranging from 1 to 7 with increasing severity^35^. As a cut-off for frailty a CFS of 4 or greater was used. To assess the burden of comorbidities we utilized the Charlson Comorbidity Index which is a scoring tool evaluating the 10-year mortality of patients with multiple comorbidities^36^.

### Temporal muscle measurement

We opted for the evaluation of temporal muscle as it is readily available from pretherapeutic imaging in neuro-oncologic patients and exhibits good correlation with other imaging assessments of sarcopenia^21,22^. One way of assessing the temporal muscle is its thickness at an axial image but methodologies vary. Because of better reproducibility we instead opted for an assessment of the temporal muscle volume^37^.

Data was collected retrospectively from routine radiographic data. Data collection was planned beforehand and was not changed during this study. Volume of the temporal muscle was calculated from the routine immediate preoperative MRI using Sectra Workstation IDS7 software Version 22.2.6.4194 (Sectra AB, Sweden). For this purpose, a T1w isotropic non contrast enhanced sequence (T1 MPRAGE) was reconstructed in triplanar 1 mm slices. Afterwards the manual volume calculation tool was utilized to outline the temporal muscle with measurements being calculated automatically. Afterwards measurements were averaged between left and right sides and independently reviewed by a second researcher. In the case of non-identical measurements cases were discussed to reach consensus.

### Statistics

For all ordinal or nominal variables absolute and relative frequencies were calculated. In case of rational scaled variables normal distribution was tested using the Shapiro-Wilk Test. The values of the normal-distributed variables were presented as the mean and standard deviation, while the values of the non-normal distributed variables were shown as the median and interquartile range. In both cases minima and maxima were calculated. Correlations were calculated using the spearman correlation coefficient. Survival was analyzed using Kaplan-Meier Curves and Cox Regression. For group comparisons we used the χ2-tests. Time-dependent receiver operating characteristic (ROC) analyses with calculation of the area under the curve (AUC) were performed in order to identify an optimal cutoff for OS and PFS. For the cut-off the maximal Youden Index (value for which sensitivity + specificity − 1) was chosen. Missing data was excluded from analysis.

Statistical analysis was performed using R: A language and environment for statistical computing R Core Team (2021) and IBM SPSS Version 26.0.0.0.

### Ethics

According to the local laws of Rhineland Palatinate, Germany (Landeskrankenhausgesetz § 37) no formal approval and informed consent was necessary for this retrospective analysis. The patient data was de-identified before analysis.

### Limitations

Our study has several inherent limitations. The retrospective design introduces a risk of bias. As assessment of frailty is a topic of ongoing debate with a multitude of tests used for classification, we decided on using broadly applied scoring systems for frailty, physical capacity and comorbidities in conjunction with temporal muscle volume.

For dichotomization we performed ROC analysis resulting in a cut-off value based on our cohort characteristics. And compared with a separate cohort suffering SAH but further external validation is needed for generalization of these results. As most other authors use their respective cohorts median TMV a universal cut-off is yet missing. The SAH cohort is of a considerably smaller size than our NSCLC cohort which is mainly due to the infrequent performance of timely MRI studies available in these patients. A short timeframe was chosen as we wanted to minimize the possibility of muscle loss after SAH to be included in our analysis.

## Results

### Baseline demographics

From 2011 to 2020 we included 179 patients in this study of whom 81 (45.3%) were women. The median age was 63 (56–69) years. At initial diagnosis AJCC Stage 1 was present in 3.93%, Stage 2 in 3.14, Stage 3 in 10.23% and Stage 4 in 82.67% of patients. Singular metastasis was present in 64 patients (35.8%) and 24 patients (13.4%) presented with infratentorial lesions. In 84 cases (46.9%) the central nervous system (CNS) was the first site of metastasis. Average time from disease diagnosis to first emergence of brain metastasis was 1 (0–13) months. PD-L1 was expressed on a median of 20% (2.25–70.25%) of the analyzed cells in histopathology. Of all patients 156 (87.2%) received radiotherapy with 85 (47.5%) receiving immunotherapy. Baseline variables are presented in Table 1.

The median Clinical Frailty Score (CFS) was 3 (3–4), classifying 37 patients (20.6%) as frail. Median Charlson Comorbidity Index (CCI) was 9 (8–10). Median Karnofsky Performance Score (KPS) before surgery was 80 (70–90), with the median post-surgery being 80 (80–90). Median TMV was 20.8 (14.2–26.7) cm^3^ with a mean of 21.1 ± 9.5 cm^3^.

To compare the TMV in our NSCLC cohort with previously healthy patients, we chose a cohort suffering from aneurysmal subarachnoid hemorrhage (SAH) as this disease has a characteristic sudden onset. Median TMV in patients suffering SAH was 16.6 cm^3^, with a mean of 18 ± 7.9 cm^3^. Functional Scores and temporal muscle volumes are presented in Table 2. Characteristics of the SAH cohort are presented in Table 3.

Median CRP was 3.9 (1.32–11.32) mg/L, median TSH was 0.78 (0.4–1.5) µIU/L, median Creatinine was 0.81 (0.73–0.99) mg/dL and median Albumin was 65 (35–74) g/L. Laboratory Values are presented in Table 4.

### Frailty, sarcopenia and age

Frailty as assessed by CFS correlated significantly with age (*r* = 0.36, *p* < 0.001) and preexisting comorbidities (*r* = 0.23 *p* = 0.014).

Volume of the temporal muscle correlated with sex, being higher in male patients (*r* = 0.46, *p* < 0.001). Additionally, TMV correlated with CFS (*r*=−0.24, *p* = 0.002) and TSH showed a significant correlation with TMV (*r* = 0.25, *p* = 0.021).

**Fig. 1:**
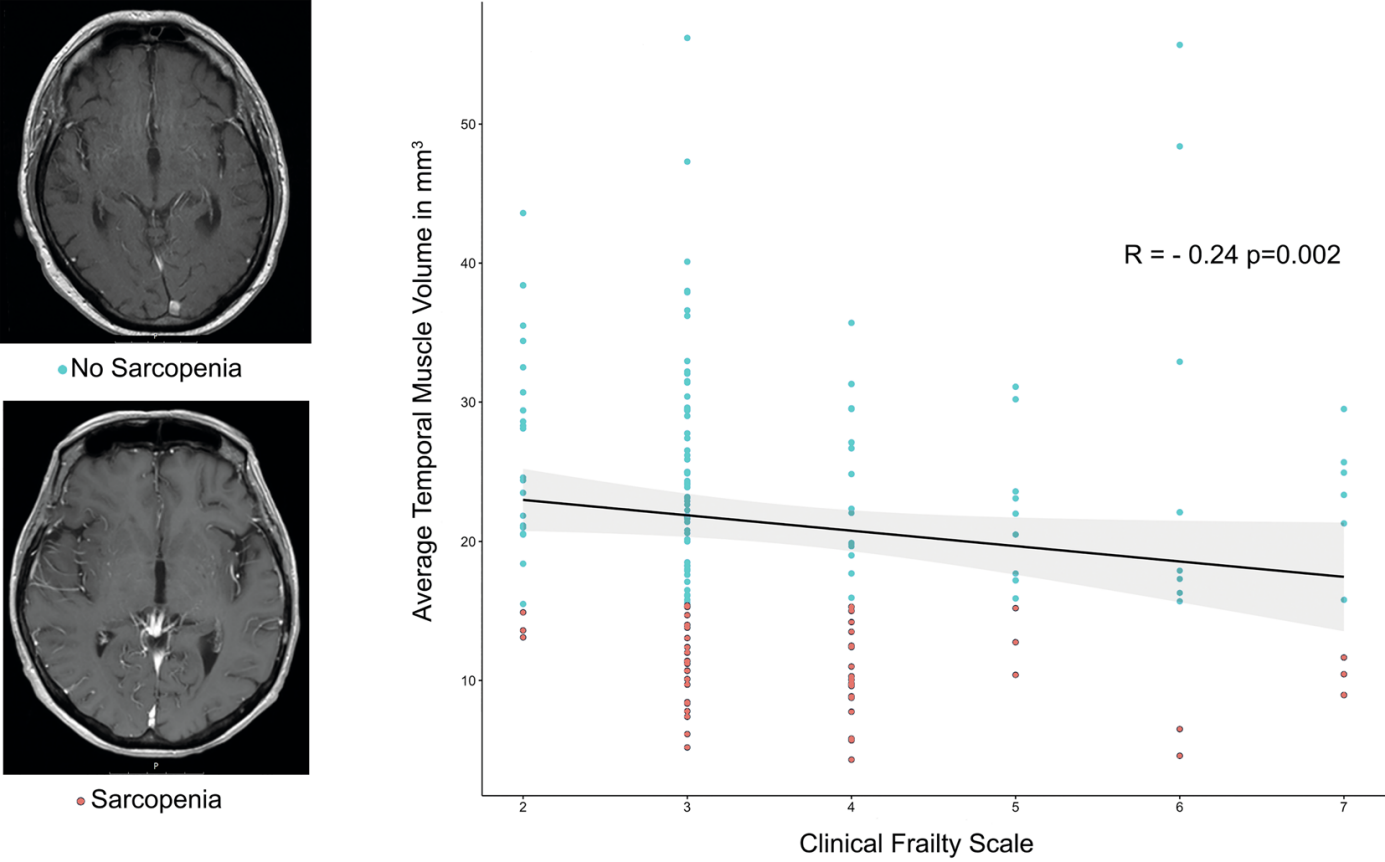
Distribution of average temporal muscle volume and clinical frailty scale. Individuals classified as sarcopenic are shown in red. (right) Exemplary axial T1 MRI images of sarcopenic and non-sarcopenic individuals. (left).

There were no correlations between CFS, TMV and age, stage, localization or number of metastases, preoperative KPS and CCI. Association of frailty and sarcopenia is displayed in Fig. 1.

In order to classify patients by TMV ROC analysis was performed for 1-year PFS and OS. Optimal cut-off in both tests was found to be 15.5 cm^3^. For further analysis patients below this cut-off were considered sarcopenic, which applied to 53 patients (29.6%). ROC Curves and AUC statistics are supplied in Supplement 1.

### Survival according to sarcopenia and frailty

Median follow-up after surgery was 11 (0–120) months with a median cerebral progression free survival (cPFS) of 8 (3 - 23) months, median systemic progression free survival (sPFS) of 13 (11 - 25) months and overall survival of 11 (8 - 34) months.

In order to assess possible influences of frailty on progression free- and overall survival (OS) patients were dichotomized according to CFS or TMV. As a cut-off to consider patients frail a CFS score of 4 was used, the cut-off in TMV to consider patients sarcopenic was below or equal to 15.5 cm^3^. Classifying patients by CFS did not show a significant difference in PFS or OS (cPFS: 24 vs. 20 months | sPFS: 13 vs. 7 months | OS: 34 vs. 29 months; *p* = 0.455 | *p* = 0.35 | *p* = 0.278).

**Fig. 2:**
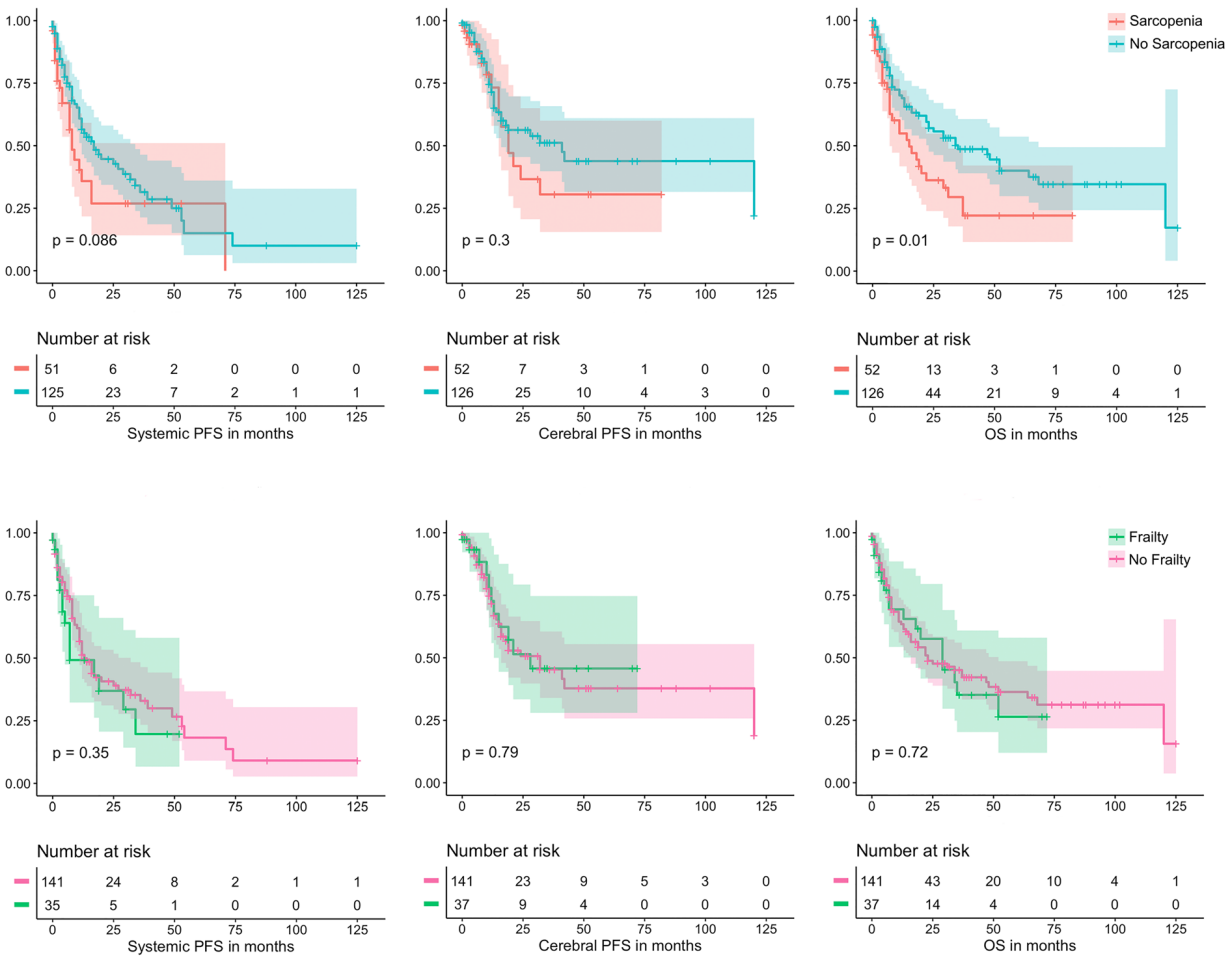
Kaplan Meier curves and risk tables for systemic/cerebral progression-free survival and overall-survival according to sarcopenia (A-C) and frailty (D-F).

Comparing patients based on sarcopenia according to the above-mentioned classification revealed a highly significant difference in median OS of 34.5 vs. 10.3 months (*p* < 0.001) but no significant difference according to cPFS (27.7 vs. 16.3 months, *p* = 0.354) or sPFS (17 vs. 8 months, *p* = 0.35). Cerebral PFS did exhibit a trend towards shorter survival when sarcopenia was present but failed to achieve statistical significance. While there was no significant difference between groups, patients that were not classified as frail or sarcopenic had a markedly longer follow-up available, suggesting possible further differences omitted from our analysis. Kaplan-Meier Curves are presented in Fig. 2.

### Response to immunotherapy

In patients receiving immunotherapy exhibited a higher therapy response as measured by PFS in patients with TMV > 15,5cm^3^ but statistical significance was not reached. (cPFS 38.3 vs. 13.5 months, *p* = 0.071 | sPFS 14 vs. 11 months, *p* = 0.94) In patients receiving radiotherapy there was no difference in cPFS (34.8 vs. 21 months, *p* = 0.476) or sPFS (16 vs. 8 months, *p* = 0.072). In both groups a higher OS was observed in patients with TMV above the cut-off. (Immunotherapy: PFS 45.9 vs. 11.6 months; radiotherapy: PFS 37.9 vs. 10.2 months both, *p* < 0.001)

### Surgical treatment

Postoperative physical performance as measured by KPS was significantly influenced by frailty according to CFS. (preoperative *r*=−0.81, postoperative *r*=−0.72, difference in *r* = 0.27, all *p* < 0.001). Sarcopenia as assessed by TMV correlated significantly with postoperative KPS (*r* = 0.2, *p* = 0.038).

The postoperative complications that were assessed included pneumonia, pulmonary embolism, surgical infection, and rebleeding. The rate of any complication was defined as the composite complication rate. Pneumonia was significantly more common in frail patients (*p* = 0.037), but rates of composite complications (*p* = 0.136), surgical infection (*p* = 0.55), pulmonary embolism (*p* = 0.84), DVT (*p* = 0.59), and rebleeding (*p* = 0.44) were not different.

Postoperative complications were more frequent in patients classified as sarcopenic (pneumonia *p* = 0.021, surgical infection *p* = 0.072, pulmonary embolism *p* = 0.035, DVT *p* = 0.025). However, there was no difference in composite complications (*p* = 0.38) or rebleeding (*p* = 0.47). Frequencies of Complications are displayed in Table 5.

### Multivariate analysis

In multivariate analysis shorter overall survival was significantly impacted by age (HR 1.1, *p* < 0.001) and sarcopenia (HR 2.0, *p* = 0.003) but not by frailty according to CFS (HR 0.8, *p* = 0.4), sex (HR 1.1, *p* = 0.6) or presence of complications (HR 1.6, *p* = 0.1). The cox regression model attained global significance with *p* <0.001. Cox regression for PFS failed to reach global significance. Forest Plots for both are displayed in Supplement 2.

Response to immune- or radiotherapy could independently be predicted by sarcopenia according to TMV (B 2.48, *p* = 0.031) but not age (B 0.08, *p* = 0.28) or frailty classified by CFS (B −0.05, *p* = 0.881).

## Discussion

We aimed to assess the influence of frailty and sarcopenia on surgical treatment and immunotherapy in patients suffering brain metastasis from NSCLC. In our analysis sarcopenia independently predicted OS and response to immunotherapy. Its presence was associated with postoperative complications and reduced postoperative physical performance possibly indicating impeded recovery of affected patients. Frailty did not predict survival but correlated with impeded physical performance and the incidence of postoperative pneumonia. In recent years awareness for age-related changes and their consequences for therapeutic decisions has greatly increased. There is a mounting body of evidence pointing towards a need for more detailed assessment of these beyond numerical age^38^. In the context of age-related changes, a range of terms have garnered increasing attention in both clinical practice and research. Frailty and sarcopenia are of exceptional importance among these, and differences and areas of overlap are present^39^. Frailty is defined as a state of increased vulnerability and susceptibility to disease resulting from aging, whereas sarcopenia is characterized by the ongoing loss of muscle mass and function. Sarcopenia constitutes a significant component of frailty; however, frailty itself is a rarer condition, and it is characterized by more profound changes^40^. In the presence of cancer frailty and sarcopenia were both shown to influence treatment efficacy and results as well as tolerability of therapeutic measures like chemo- or immunotherapy^23,28,41,42^. Additionally neoplastic disease can increase muscle loss and further strain limited resources in the affected individual^39^.

Improvements in neuroimaging, and better prognosis through more effective systemic therapies lead to earlier and more frequent detection of CNS dissemination of systemic tumors^43^. Additionally, tumor incidence increases with age. Given the physical demands of multimodal therapy, prognostic markers and assessment tools for age-related changes are necessary to optimize individualized treatment. Multiple studies demonstrate the importance of assessing frailty when determining oncologic treatment^44^.

In the presented cohort, frailty according to CFS was present in 20.6% of all patients with NSCLC. In a meta-analysis the prevalence of frailty was reported to be as high as 45% of patients suffering from NSCLC^13^. The reported prevalence is higher, compared to the presented data and other literature. Frailty in a uniform cohort of patients with brain metastasis from NSCLC was found to be present in 24%.^24^ This proportion fits the prevalence of frailty in our presented cohort. Differences in frailty between patients with NSCLC per se and those with brain metastasis might be explained by differences in age or clinical performance status as CNS invasion occurs more often in later stages of the disease after longer previous periods of therapy^3,45^. Another possible explanation for the lower frailty rates in patients receiving surgical treatment for brain metastasis of NSCLC might be preselection and therapeutic bias by the treating physicians, leading to exclusion of patients that could potentially benefit from surgical treatment^24^. To prevent treatment disparity, more independent prognostic markers for patient stratification other than patients’ age and physician estimation, are needed.

Since frailty is a clinical state influenced by various age-related changes, it often occurs alongside other conditions. In our study frailty correlated with age, comorbidities, physical performance status as well as sarcopenia. As frailty is defined as a state of increased risk for adverse health outcomes that is determined by multiple factors and is associated with age, the observation in the presented cohort aligns with previous findings^46^. However, there was no independent correlation between frailty and survival in the presented cohort. This stands in contrast to previously published data where frailty is associated with all-cause mortality and specific mortality among cancer patients^44^. Prognostic relevance of frailty is established in different entities including glioma, colorectal carcinoma and NSCLC^11,13,24,47^. One potential contributing factor is the absence of a universally applicable assessment tools for frailty. Therefore, studies employ a variety of methodologies to identify patients afflicted with the condition, thereby accounting for potential discrepancies in data comparison^13,24,48^. Moreover, frailty evaluation frequently involves a certain degree of subjectivity, further complicating the comparison of findings^49^. An additional factor that may be considered is the relatively young age of the subjects in our cohort. Ilic et al. did not observe a prediction of survival by utilizing the modified Frailty Index in patients younger than 65 years of age who suffered from brain metastasis of non-small cell lung cancer (NSCLC)^24^. Yet in the broader oncologic literature frailty is to be considered a relevant factor for survival despite our observations^13^. However, differences in frailty assessment and spatial severity render frailty a particular hard to standardize and potentially bias-afflicted clinical parameter. Muscle loss is an important contributor to frailty and promises to add reproducibility in frailty assessment as it is less prone to fluctuation and interobserver variance^24^.

Retrospective measurements of sarcopenia frequently rely on available imaging as a standard using whole body- or lumbar paravertebral musculature as surrogate parameters^20^. Especially in oncologic studies imaging analyses are widely accepted as measurements of muscle strength might be confounded by cachexia caused by neoplastic disease^39^. The most relevant measurement in oncologic studies is the paravertebral muscle area at the L3 level^39^. As radiographic whole-body measurements aren’t necessarily available through standard diagnostics in the setting of neurosurgical treatment, temporal muscle volume is widely used and correlates well with measurements of body composition and frailty^21^. While some authors recommend assessment of sarcopenia to include muscle quality in oncologic studies muscle mass analysis is commonly used alone, as strength measurements can be impeded by tumor related cachexia which seems to be less relevant for muscle mass evaluations^39^. For this study temporal muscle was used because of its availability from preoperative cranial MRI. When assessing the temporal muscle, measuring muscle thickness and volume is commonly used. As measurement of TMT reduces a three-dimensional structure to a two-dimensional representation, we decided on volumetric analysis to possibly achieve more accurate representation of muscle mass. Measurement of TMT is performed parallel oriented to the anterior-posterior commissure line with the sylvian fissure and the orbital roof as anatomical landmarks^26^. To define sarcopenia in our cohort we performed ROC analysis for 1-year PFS and OS, 15.5cm^3^ were identified as cut-off value. In literature, the median temporal muscle thickness is often used for differentiation, yet there is a lack of evidence and broader applicability for those classification^20,50^. In the presented cohort, determination of individual AUC threshold values proved statistically superior with better estimation and potentially offers insight for future, disease specific, standardization. To validate the calculated TMV cut-off value and comparison with previously healthy patients suffering from aneurysmal subarachnoid hemorrhage was performed. Due to the apoplectic onset of SAH those patients provide evidence of baseline TMV to differentiate sarcopenia from the influence of cachexia during the course of malignant disease^51^. When comparing these cohorts the higher percentage of female patients in the SAH cohort needs to be noted which might reduce the temporal muscle mass in this cohort due to known differences between genders^52^. The calculated cut-off of 15.5cm^3^ is reasonably close to the median TMV of 16.6cm^3^ within the SAH cohort and well within the standard deviation of TMV in both cohorts. A similar cohort of patients with spontaneous SAH (median age 61) had a baseline TMV of 18.5cm^3^, where a TMV of 15cm^3^ and less, was associated with impaired functional outcome^53^. This observation is transferable to the presented Cohort of patients with brain metastasis in NSCLC and potentially argues for a more absolute state of sarcopenia to determine functional outcome across different disease.

In the presented dataset, sarcopenia independently predicts overall survival. A recent meta-analysis including 1675 patients suffering from brain metastasis of different entities found shortened PFS by sarcopenia^54^. Our observation is in line with these results. However, only studies utilizing lumbar paravertebral muscle mass were included in this meta-analysis. Since the applied measurement technique differ, we think this further supports the broader significance of sarcopenia. The interrelation of sarcopenia with age currently remains less clear in the setting of brain metastasis. In our multivariate analysis we observed a greater independent impact of sarcopenia (HR 2 *p* = 0.003) than age (HR 1.1 *p* < 0.001) on OS. Yet there is conflicting data in this regard. There are studies which only observed a relationship between sarcopenia and survival only in patients older than 65 years suffering from brain metastasis of NSCLC, while others observed a similar influence irrespective of age groups but found age to be another independent predictor of survival^24,55^. Evaluation of sarcopenia as a predicting factor on progression free survival in patients receiving immunotherapy showed a tendency towards increased PFS in Kaplan Meier analysis. There was no such trend noted in patients receiving radiotherapy. These results are consistent with previous findings on immunotherapy in advanced NSCLC^56,57^. Possible explanations for these observations are decreased immunoreactivity thus decreasing antitumoral immune activity and decreased endocrine function of muscle tissue in sarcopenia promoting dysregulation of immunoresponse^58,59^. To our knowledge our results are the first from patients receiving surgical treatment for brain metastasis considering response to consecutive immunotherapy.

Considering our observation that sarcopenia but not frailty predicted survival in our cohort a certain conflict with established literature is to be addressed. Available data suggest that both, frailty and sarcopenia are risk factors of decreased progression-free and overall survival in patients suffering brain metastases irrespective of primary tumor type^24,42,55^. Possible explanations for these results include the relatively young median age in our cohort and comparatively low rates of frailty in comparison to other studies evaluating patients suffering brain metastasis^24,42^. This might be a reflection of potential selection by involved physicians or could potentially be based in some degree of subjectivity in the applied screening tools for frailty^49^. Another reason may be the prevalence of age-related treatment bias, which makes frailty analysis susceptible to misinterpretation. We believe that this issue should be emphasized in the highlighting of recent studies, as frailty is now increasingly recognized in clinical routine.

Reflecting the currently available data on frailty and sarcopenia in conjunction with the presented findings, sarcopenia is an independent predictor of survival in patients suffering from brain metastases of NSCLC. While we did not observe a significant influence of frailty, the available literature supports a relevant influence of frailty on survival in afflicted patients.

## Conclusion

Sarcopenia is an independent predictor of OS and predicts response to immunotherapy better than frailty or age. Additionally, sarcopenia impedes postoperative physical performance and increases complication rates. Sarcopenia should therefore be accounted for in therapeutic and surgical decision making to improve patient selection for different treatment modalities.

**Table 1:** – Demographics.

	Patients with NSCLC
N	179
Sex	
Female	81 (45.3%)
Age [a, 1.Q, 3.Q]	63 (56–69)
Localization	
Infratentorial	24 (13.4%)
Singular Metastasis	64 (35.8%)
Primary Metastasis	84 (46.9%)
Time to Metastasis [m, Range]	1 (0–13)
PD 1/PD-L1 Expression [%; range]	20 (2.25–70.25)
Radiotherapy	156 (87.2%)
Immunotherapy	85 (47.5%)
Follow up [m, Range]	11 (0–120)
PFS [M, 1.Q, 3.Q]	8 (3 - 23)
OS [M, 1.Q, 3.Q]	11 (8-34)

A: Years, M: Months, PFS: Progression Free Survival, OS: Overall Survival, 1.Q: 1. Quartile, 3.Q: 3. Quartile.

**Table 2:** Functional Scores.

CFS [1.Q, 3.Q]	3 (3–4)
Frailty by CFS	37 (20.6%)
CCI [1.Q, 3.Q]	9 (8–10)
TMV [cm^3^, 1.Q, 3.Q]	20.8 (14.2–26.7)
TMV above Median	89 (49.7%)
Sarcopenia by TMV cut-off	53 (29.6%)
KPS Pre [1.Q, 3.Q]	80 (70–90)
KPS Post [1.Q, 3.Q]	80 (80–90)
ΔKPS [1.Q, 3.Q]	0 (0–10)

CFS: Clinical Frailty Scale, CCI: Charlston Comorbidity Index, TMV: Temporal Muscle Volume, CM^3^: Cubic Centimeters, KPS: Karnofsky Performance Score, 1.Q: 1. Quartile, 3.Q: 3. Quartile.

**Table 3:** Baseline comparison SAH.

	Controls	Metastasis
N	31	179
Female	23 (71.8%)	81 (45.3%)
Age [a, 1.Q, 3.Q]	58 (55–62.5)	63 (56–69)
CFS [1.Q, 3.Q]	3 (2–4)	3 (3–4)
CCI [1.Q, 3.Q]	2 (1–2)	9 (8–10)
TMV [cm^3^, 1.Q, 3.Q]	16.6 (12–21.1)	20.8 (14.2–26.7)

CFS: Clinical Frailty Scale, CCI: Charlston Comorbidity Index, TMV: Temporal Muscle Volume, CM^3^: Cubic Centimeters.

**Table 4:** Laboratory Values.

CRP [mg/L, 1.Q, 3.Q]	3.9 (1.32–11.32)
TSH [µIU/L, 1.Q, 3.Q]	0.78 (0.4–1.5)
Creatinine [mg/dl, 1.Q, 3.Q]	0.81 (0.73–0.99)
Albumin [g/L, 1.Q, 3.Q]	65 (35–74)

CRP: C-reactive Protein, TSH: Thyroid Stimulating Hormone, MG/L: Milligram per Liter, µIU/L: Mikro International Units per Liter, mg/dl: Milligram per Deciliter, g/dL: Gram per Deciliter.

**Table 5:** Complications.

	All	Frailty	Sarcopenia
Combined	25 (13.9%)	8 (21.6%)	19 (15.1%)
Pneumonia	11 (6.1%)	5 (13.5%)	8 (6.3%)
Surgical Infection	8 (4.5%)	1 (2.7%)	6 (4.8%)
DVT	3 (1.7%)	1 (2.7%)	2 (1.6%)
PE	6 (3.4%)	1 (2.7%)	3 (2.4%)
Rebleeding	6 (3.4%)	2 (5.4%)	5 (4.0%)

DVT: Deep Vein Thrombosis, PE; Pulmonary Embolism.

### Data Availability

The datasets generated during and analyzed during the current study are available from the corresponding author on reasonable request.
