# Correction: Association of sarcopenia with survival and treatment response in brain metastasis of non-small cell lung cancer

**DOI:** 10.1038/s41598-026-58620-w

**Published:** 2026-06-22

**Authors:** Leon Schmidt, Harald Krenzlin, Anika Schmitz, Dragan Jankovic, Alice Dauth, Beat Alessandri, Clemens Sommer, Marc A. Brockmann, Florian Ringel, Naureen Keric

**Affiliations:** 1 Department of Neurosurgery, University Medical Center Mainz, Mainz, Germany; 2 Department of Neurosurgery, University Hospital Schleswig-Holstein (UKSH), Ratzeburger Allee 160, 23538 Lübeck, Germany; 3 Institute of Neurpathology, University Medical Center Mainz, Mainz, Germany; 4 Department of Neuroradiology, University Medical Center Mainz, Mainz, Germany

Correction to: *Scientific Reports* 10.1038/s41598-026-37138-1, published online 05 February 2026

The original version of this Article contained error in the name of the author Marc A. Brockmann, which was incorrectly given as Marc A. Brockman.

The original Article has been corrected.

